# Quality indicators for hospital burn care: a scoping review

**DOI:** 10.1186/s12913-024-10980-7

**Published:** 2024-04-19

**Authors:** Denise R. Rabelo Suzuki, Levy Aniceto Santana, Juliana Elvira H. Guerra Ávila, Fábio Ferreira Amorim, Guilherme Pacheco Modesto, Leila Bernarda Donato Gottems, Vinicius Maldaner

**Affiliations:** 1 Programa de Pós-Graduação em Ciências para a Saúde, Escola Superior de Ciências da Saúde (ESCS/FEPECS), SMNH Quadra 3 Conjunto A Bloco 01 Edifício Fepecs, Asa Norte, Brasília, Distrito Federal Brazil; 2 Secretaria de Saúde do Distrito Federal (SES-DF), Setor de Rádio e TV Norte (SRTVN) 701, Via W5 Norte, lote D, Brasília, Distrito Federal Brazil; 3 Programa de Pós-Graduação em Ciências da Saúde, Universidade de Brasília (UnB), Campus Universitário Darcy Ribeiro, Asa Norte, Brasília, Distrito Federal Brazil; 4 Programa de Pós Graduação em Ciências do Movimento Humano e Reabilitação, Universidade Evangélica de Goiás, Cidade Universitária, Avenida Universitária, Anápolis, Goiás Brazil; 5 Universidade de Brasília (UnB), Ceilândia Sul Campus Universitário, Centro Metropolitano, Ceilândia, Distrito Federal Brazil; 6 Unidade de Queimados, Hospital Regional da Asa Norte (HRAN), 3° andar. Setor Médico Hospitalar Norte Q 2, Brasília, Distrito Federal 70710-100 Brazil

**Keywords:** Burns, Quality indicators, health care, Health care quality, access and evaluation, Hospital care

## Abstract

**Background:**

Burn treatments are complex, and for this reason, a specialised multidisciplinary approach is recommended. Evaluating the quality of care provided to acute burn patients through quality indicators makes it possible to develop and implement measures aiming at better results. There is a lack of information on which indicators to evaluate care in burn patients. The purpose of this scoping review was to identify a list of quality indicators used to evaluate the quality of hospital care provided to acute burn patients and indicate possible aspects of care that do not have specific indicators in the literature.

**Method:**

A comprehensive scoping review (PRISMA-ScR) was conducted in four databases (PubMed, Cochrane Library, Embase, and Lilacs/VHL) between July 25 and 30, 2022 and redone on October 6, 2022. Potentially relevant articles were evaluated for eligibility. General data and the identified quality indicators were collected for each included article. Each indicator was classified as a structure, process, or outcome indicator.

**Results:**

A total of 1548 studies were identified, 82 were included, and their reference lists were searched, adding 19 more publications. Thus, data were collected from 101 studies. This review identified eight structure quality indicators, 72 process indicators, and 19 outcome indicators listed and subdivided according to their objectives.

**Conclusion:**

This study obtained a list of quality indicators already used to monitor and evaluate the hospital care of acute burn patients. These indicators may be useful for further research or implementation in quality improvement programs.

**Trial Registration:**

Protocol was registered on the Open Science Framework platform on June 27, 2022 (https://doi.org/10.17605/OSF.IO/NAW85).

**Supplementary Information:**

The online version contains supplementary material available at 10.1186/s12913-024-10980-7.

## Background

According to the World Health Organization (WHO), burns are a public health problem worldwide, accounting for about 180,000 deaths annually, mostly in low- and middle-income countries [1]. In addition to the mortality impact, burns can present devastating results for the individual's health and are associated with expensive and prolonged hospitalisation and rehabilitation programs, with substantial losses in quality of life [2, 3]. Despite the important advances in recent decades, it is currently recommended that these patients be treated by a specialised multidisciplinary team [2].

Quality indicators are increasingly being used in health services worldwide. They consist of measurement tools, usually based on standards of care, aiming to monitor performance, inform policies or strategies, and support improvements in clinical practice [2, 4]. An important step in the process of evaluating the quality of health care is the identification of appropriate indicators. Each indicator will reflect different aspects of quality, and its selection will depend, among others, on the objectives of the analysis, the data available, and for whom it is intended [5].

In the context of burns, infection control, fluid management, and wound treatment, among other factors, are critical for the patient's outcome and survival. Evaluating the quality of care provided to these patients enables the development and implementation of measures to help improve the standard of care and the results achieved [6].

In the literature, it is possible to find studies describing the process and results of developing a list of quality indicators aimed at the care of burns, generally developed through a consensus among experts [2, 4, 7–9]. However, none of these instruments are specific to evaluate hospital care. Data demonstrate that hospital-acquired events can further impact the patient's long-term quality of life with burn sequelae [10]. Obtaining a list of indicators that apply to this delicate phase of care enables better practices and, consequently, better results.

The objective of this scoping review is to answer the question: "What indicators are used to evaluate the quality of hospital care provided to acute burns patients?". It aims to identify and obtain a list and indicate possible aspects of care that do not have specific indicators in the literature. It is part of a larger project that aims to build an instrument to evaluate the quality of care provided by Brazilian burn units.

## Methods

A scoping review was performed following the PRISMA Extensions for Scoping Reviews (PRISMA-ScR) guidelines [11], conducted according to a protocol registered on the Open Science Framework [12] platform on June 27, 2022, and can be accessed at https://doi.org/10.17605/OSF.IO/NAW85.

### Research strategy

The search was performed between July 25 and 30, 2022, and redone on October 6, 2022, in the following databases: PubMed (MEDLINE), Cochrane Library, EMBASE, Lilacs/VHL.

The search strategy was developed by one of the researchers (DS) and reviewed by two others (VS and LAS). The search strategy was formulated for MEDLINE (Additional_File 1) and adapted for the other databases, using the following descriptors and their respective synonyms: ((Burns[Title/Abstract]) OR (Burns, Inhalation[Title/Abstract]) OR (Smoke Inhalation Injury[Title/Abstract]) OR (Burns, Electric[Title/Abstract]) OR (Burns, Chemical[Title/Abstract])) AND ((Quality Assurance, Health Care[Title/Abstract]) OR (Quality Improvement[Title/Abstract]) OR (Quality Indicators, Health Care[Title/Abstract]) OR (Health Care Quality, Access[Title/Abstract] AND Evaluation[Title/Abstract]) OR (Health Care Evaluation Mechanisms[Title/Abstract]) OR (Patient Reported Outcome Measures[Title/Abstract]) OR (Outcome[Title/Abstract] OR (Quality of Health Care[Title/Abstract])).

In addition, references from included studies were searched manually to identify potential additional studies.

### Eligibility criteria

Potentially relevant articles were evaluated for eligibility based on inclusion and exclusion criteria following the PCC guide:

- Population – acute burn patients
- Concept – quality indicators

- Context – hospital care

Original or review studies published in indexed journals and documents in governmental or specialised societies were included, addressing acute burn patients (open wounds), without distinction of age, sex, or causal agent, studies presenting quality of care measurement, and managing patients hospitalised for burn treatment. There were no restrictions on the length of time or language of publication. Studies that did not address burns or quality indicators or addressed burned patients with wounds already healed or in pre-hospital, emergency room, outpatient intervention, or rehabilitation phase were excluded.

As this review is the initial stage of a larger study that aims to develop an instrument to evaluate the quality of care in Brazil, studies that explicitly presented quality indicators and constructs to be considered from a quality perspective were selected.

### Screening procedures and data extraction

The references found were organised using the Rayyan Platform [13] and analysed for eligibility by two independent reviewers (DS and JA). In cases of disagreement, a third reviewer was consulted (VS). The reasons for exclusion from the studies were recorded.

General data such as title, authors, journal, year and language of publication, country where it was performed, study design, and data on the identified quality indicators were collected. Each indicator was classified as structure, process, and outcome, as proposed by Donabedian [14]. In addition, information on the indicator's purpose and how it was calculated was also collected. The data were extracted through a standardised form developed in Microsoft Word (Additional_File 2).

After data extraction, each indicator was organised into a list of structure, process, and outcome indicators. The structure indicators were divided, according to their objective, into indicators to evaluate the physical structure, human and organisational resources. The process indicators were distributed into indicators to evaluate, the treatment of the burned patient, prophylactic measures, complications, and other process indicators. Finally, the outcome indicators were categorised to evaluate mortality, length of hospital stay, wound healing, physical, functional and nutritional results, and other outcome indicators.

In addition, a table was organised containing the main characteristics of each article and the indicators identified (Additional_File 3).

## Results

**Fig. 1:**
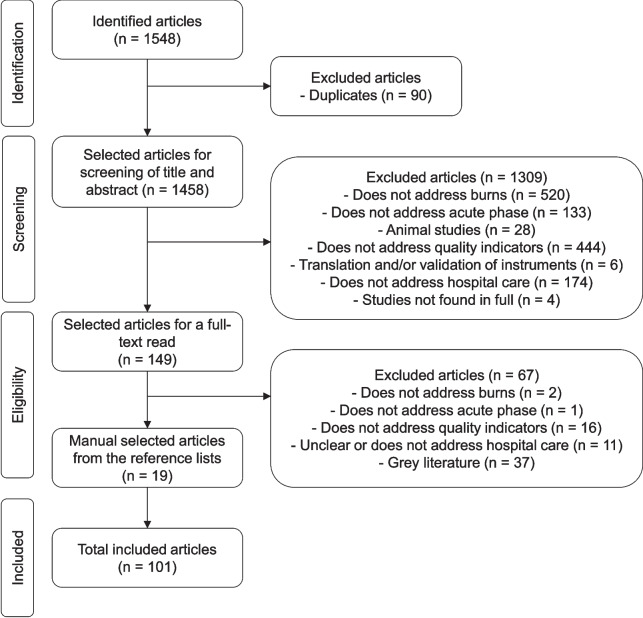
PRISMA flowchart

A total of 1548 studies were identified, and after removing the duplicates, 1458 publications were included in the screening process. Among these, 82 were included, and their reference list was searched, adding 19 more, totalling 101 studies. The identification and selection process are shown in Fig. 1.

The main characteristics and the quality indicators identified in each study can be seen in Additional_File 3. The most common study design was a retrospective cohort (*n* = 33, 32.67%), followed by randomised controlled trials (*n* = 14, 13.86%). Most of the studies were published starting from 2000 (*n* = 97, 96%), and most were published in the last ten years, between 2013 and 2022 (*n* = 77, 76.23%). Regarding the journal of publication, most studies were found in specific burn journals (*n* = 63, 62.37%), and the most used language was English (*n* = 76, 75.24%). Most of the studies were conducted by American institutions (*n* = 41, 40.59%), and two were performed with other countries (Canada and Israel).

Tables 1, 2, and 3 present a list of all quality indicators and constructors identified and the studies in which they were found.

**Table 1:** Structural quality indicators

**Physical structure**
1. Adequate structure for hand hygiene [15]
**Human resources**
2. Presence of a surgeon in the burn unit 24 h a day [4]
3. Multidisciplinary care [4]
**Organisational resources**
4. Multidisciplinary team weekly meetings [4]
5. Admission protocol in place [16]
6. Care protocol in place [16]
7. Involvement in teaching [16]
8. Maximum capacity of hospitalisation respected [16]

**Table 2:** Process quality indicators

**Burn patient assessment**
1. Inhalation injury assessment [9, 17–20]
2. Evaluation by surgeon/nurse within 24 h after admission [4]
3. Total body surface area (TBSA) assessment [2, 9, 20, 21]
4. Evaluation of other wound characteristics in addition to the TBSA [16, 20, 21]
5. Mortality risk assessment [22–27]
6. Time of physical-functional assessment [2, 4, 9, 20]
7. Weight and height assessment on admission and during hospitalisation [4, 28–30]
8. Calculation of Body Mass Index (BMI) [29, 31, 32]
9. Malnutrition risk assessment and nutritional assessment [2, 30]
10. Indication of artificial feeding [28, 30]
11. Time of psychosocial assessment [2, 8, 9]
12. Pain assessment on admission and during hospitalisation [2, 33–43]
13. Nurse's perception of pain [43]
14. Surveillance of infection at admission [4]
15. Evaluation of pruritis during hospitalisation [7, 33]
**Burn patient treatment**
16. Need for decompression procedure [2, 9, 20, 44, 45]
17. Estimation of resuscitation fluid volume [2, 9, 46]
18. Elapsed time from injury to initiate resuscitation [19]
19. Resuscitation fluid volume and urinary output [16, 20, 46–48]
20. Albumin use [49, 50]
21. Monitoring of resuscitation volume [9, 46, 48, 51]
22. Time from admission to start feeding [2, 4, 8, 9, 28, 29, 52, 53]
23. Energy and protein needs [7, 28–30, 32, 54, 55]
24. Total calories offered and ingested by the patient [7, 9, 20, 28–32, 54–56]
25. Use of glutamine and oxandrolone [9, 20]
26. Diet markers [9, 20, 29–31, 54, 55, 57, 58]
27. Micronutrient supplementation [30, 54]
28. Duration of hydrotherapy in minutes [43]
29. Total number of dressings during hospitalisation [59]
30. Total number of surgical procedures [35, 60–64]
31. Duration of surgical procedures [16, 61, 62, 65–68]
32. Time from admission to first surgical excision [4, 8, 19]
33. Time to complete eschar removal [2, 4, 9, 20, 44, 45, 67]
34. Incidence and percentage area of excised wounds treated surgically [44, 45]
35. Days from injury to first grafting and from first to last grafting [16, 59]
36. Number of grafting procedures and percentage of wound grafted [16, 40, 44, 45, 59, 60, 69]
37. Patient/caregiver understanding of the surgical procedure [7]
38. Patient/caregiver satisfaction with the postoperative period [7]
39. Pain management [7, 8, 34, 36, 37, 70]
40. Duration of rehabilitation in days [71]
41. Rest time in bed [71]
42. Registration of mobility level [72]
43. Palliative care assessment [2]
**Prophylactic measures**
44. Anticoagulation prophylaxis [2]
45. Hand hygiene adherence rate [15, 73]
46. Preventive measures for central venous catheter-associated infections [73]
47. Preventive measures for ventilator-associated pneumonia (VAP) [73]
**Complications**
48. Complications of resuscitation volume [7, 46, 48]
49. Positive blood cultures and pathogen identification [2, 18]
50. Incidence of healthcare-associated infections [7, 9, 18, 20, 35, 40, 52, 53, 56, 58, 59, 63, 66, 68, 73–78]
51. Incidence and duration of sepsis [7, 19, 52, 62, 66, 74]
52. Incidence of renal complications [4, 19, 52]
53. Unplanned extubation rate [79]
54. Incidence of organ dysfunction [48, 53, 77]
55. Readmission to the Intensive Care Unit (ICU) [2]
56. Incidence of decubitus ulcers/maintenance of the integrity of unburned skin [7, 80]
57. Diet-related complications [55, 56]
58. Incidence of bronchoalveolar aspiration [56]
59. Incidence of perioperative hypothermia [66, 81]
60. Need for re-grafting [9, 20, 68, 74]
61. Blood loss during surgery [44, 45, 62, 68, 78, 82, 83]
62. Rehabilitation-related complications [84]
63. Anxiety assessment [7, 36, 39, 42, 85, 86]
64. Depression assessment [9, 20, 85]
65. Acute stress disorder assessment [9, 20]
66. Self-esteem assessment [85]
67. Sleep quality assessment [7, 36, 86]
68. Monitoring of hypermetabolism [7, 29, 57]
69. Other complications [19, 30, 33, 41, 63, 66, 74, 87]
**Other process indicators**
70. Nursing empathy with the burn patient [88]
71. Patient/caregiver understanding of post-hospital care [7]
72. Resolvability [60]

**Table 3:** Outcome quality indicators

**Mortality**
1. Gross mortality and standardised mortality [4, 9, 16–19, 21, 24, 26, 27, 29, 30, 49, 50, 52, 53, 55, 56, 60, 62, 64, 65, 74, 77, 84, 87, 89, 90]
2. Lethal area 50 (LA-50^a^) [17, 20, 91]
**Length of hospital stay**
3. Length of hospital stay [4, 16, 18, 21, 23, 26, 35, 38, 40, 49, 50, 52, 55–62, 64, 65, 68, 69, 71, 74, 78, 84, 89, 90, 92–97]
4. Length of ICU stay [4, 17, 26, 46, 52, 53, 56, 65, 71, 84, 90, 98]
5. Length of hospital stay / total body surface area [20, 27, 30, 63, 99]
6. Number of days on mechanical ventilation [4, 17, 46, 53, 63, 65, 71, 74, 84]
**Wound healing**
7. Graft adhesion percentage [7, 8, 78]
8. Healing time of the donor area [7, 41, 57]
9. Time to wound closure/ percentage of wound healed at discharge [7, 9, 20, 33, 35, 38, 40, 44, 45, 62, 100]
**Physical-functional outcomes**
10. Evaluation of range of motion [7, 20, 71, 101–103]
11. Incidence of ectropion, microstomia, and nasolabial contractures [102]
12. Muscle strength assessment [7, 20, 72]
13. Functionality assessment [7, 71]
14. Distance the patient can walk at discharge [7, 61, 72, 98]
15. Evaluation of pneumo-functional results [7, 104]
**Nutritional outcomes**
16. Weight loss during hospitalisation [4, 7, 9, 20, 28, 54, 55, 57, 98]
**Other result indicators**
17. Quality of life assessment [9, 20, 89, 105–107]
18. Assessment of patient/care satisfaction [35, 37, 40, 88, 108, 109]
19. Unplanned readmission [2, 4, 110]

^a^LA50 – Total body surface area with 50% mortality

Considering that quality indicators provide a quantitative basis that can be used to monitor and evaluate the quality of care provided [111], all the indicators that were explicitly addressed in the included studies are described in Additional_File 4.

## Discussion

The main objective of this study was to identify and generate a list of quality indicators used to evaluate the quality of hospital care provided to patients with acute burns and indicate the gaps in the current literature. This review identified eight structure quality indicators, 72 process indicators, and 19 outcome indicators. Most included studies (76.23%) were published in the last ten years, demonstrating an increasing trend in assessing healthcare quality [2, 4]. Despite many indicators found, there is still a need for more detail regarding the structure indicated for quality care and a lack of evaluation of some aspects, such as speech therapy.

The structure indicators were addressed only in three studies, showing that they are still little explored in the current literature and that most are related to organisational aspects.

Regarding the physical structure of burn units, it is important to highlight that burn patients have unique characteristics and needs, and the units should be designed to address this specific care. Assuming that burn units follow the same standards as general hospitals can result in major deficiencies. A review study that sought to establish the main characteristics of the burn unit design that make it possible to provide best practices found some clinical evidence to support the configuration in closed units, with individual rooms and incorporating ICU capacity for burns [112]. The American Burn Association (ABA) places as one of the criteria for certification of a burn centre that the hospital maintains a specialised unit dedicated to caring for burn patients and has designated beds with the capacity for intensive treatment [113]. The European Burns Association (EBA), in its guideline published in 2017, states that treatment offered in specialised centres brings better results and recommends that they have adequate space, be located within a hospital equipped for all aspects of treatment and include a medical and administrative team dedicated to care and with a high level of specialisation [114]. However, despite the recommendations, there are few structure indicators, leading to a poor evaluation of the necessary structure to achieve good results.

Only a full-time specialised surgeon was highlighted regarding human resources, and a multidisciplinary team provides care. The International Society for Burn Injuries (ISBI) [115] and the EBA guidelines [114], as well as the ABA certification lists [113], recommend that a highly specialised multidisciplinary team provide treatment. In this review, no details were provided on the minimum composition of the team, and no indicators related to the nursing team were identified. Studies performed in New Zealand, Canada, and the United States observed that restructuring the nursing workforce to reduce costs significantly influenced the increase in adverse events, morbidity, and mortality of hospitalised patients [116, 117]. These studies were not specific to burn patients but reflect the need for further research to evaluate the impact that the availability of certain human resources may have on the outcomes of burn patients. The ISBI states that the multidisciplinary team should comprise at least burn surgeons, trained nurses, physiotherapists, occupational therapists, pharmacists, and nutritionists. However, depending on the complexity of the cases, they could benefit from including other professionals [115].

As for the process indicators, an extensive list was identified, corroborating the complexity of care for these patients. Pain evaluation was the most verified indicator among the 15 indicators for evaluating the burned patient. These indicators were identified in 33 studies. Burn patients invariably suffer from pain, one of the main problems for the patient and the treatment team [39]. In addition to the pain associated with the initial trauma, there is also pain related to the treatment itself, such as dressing changes, surgeries, and physiotherapy [43], extremely painful procedures that justify the importance of a specific indicator to evaluate the pain and the results obtained with the control measures adopted.

Twenty-eight indicators related to the treatment of burn patients were found in 48 articles. Nine indicators are related to surgical care, six to nutritional care, and five to resuscitation fluid.

Regarding surgical care, a systematic review with meta-analysis to evaluate the efficacy and safety of early burn excision demonstrated that this practice significantly reduced mortality (in patients without inhalation injury) and length of hospital stay [62]. Another prospective cohort study observed a lower incidence of positive cultures, better graft adherence, and an important reduction in hospital stay in patients submitted to early excision and grafting [78]. The ideal time for early excisions remains under debate; however, it is widely accepted that an adequate surgical intervention interferes considerably with the final results obtained, justifying the importance of various indicators to evaluate this aspect of care.

Severe burns result in hypercatabolic syndrome, which can persist for up to two years after injury [118]. These patients have significant energy needs and are often not able to achieve their macro and micronutrient demands orally [119]. While providing nutrition is essential and widely accepted for successful management, there are several conflicts over the best method and timing of enteral nutritional support. A systematic review conducted to evaluate the effectiveness of early vs late enteral nutritional management in burn adults demonstrated some promising results suggesting early nutritional support can attenuate the hypermetabolic response to thermal injury. Still, it was insufficient to indicate benefits in clinical outcomes such as length of hospital stay and mortality [119]. Despite this result, the early onset of nutritional support is a key aspect of managing critically ill burn patients. It is widely cited in clinical practice guidelines [120]. These data highlight the importance of indicators to assess the adequacy of the nutritional care offered to the burned patient and enable better assessments of the impact of this care on outcomes.

Most deaths occurring within 72 h of injury are caused by volume shock associated with burns. Aggressive resuscitation volume is adopted to achieve and maintain the perfusion of target organs in the face of extensive fluid losses by the burned area and fluid load in injured tissues [121]. However, excessive resuscitation can be as dangerous as insufficient resuscitation. Excessive fluid administration further increases capillary permeability, worsening fluid creep, and can lead to devastating complications such as acute respiratory distress syndrome (ARDS), congestive heart failure, abdominal compartment syndrome, and compartment limb syndrome, among others [120]. For these reasons, indicators are important to guide and monitor this initial and crucial phase of treating a critical burn.

Also related to process indicators, 22 quality indicators were found and cited in 46 articles regarding the possible complications of a burn patient. Among the 22 indicators, five are related to psychosocial complications, three are directed to infectious complications, and another three are intended to evaluate surgical complications.

Burns not only have a physical impact but can also affect the patient's psychological and emotional well-being. In addition to an often traumatising event, hospitalisation and subsequent wound treatment are painful and invasive. A systematic review conducted to evaluate the psychological impact on children's mental health after burns observed that there seems to be evidence of high risk for mental health diagnoses, in particular, diagnoses such as anxiety disorders, post-traumatic stress disorders, acute stress disorder, depression, and personality disorders [122]. Most participants in the reviewed studies experienced increased anxiety and other psychological symptoms after a burn compared to the general population [122]. These aspects demonstrate the importance of including quality indicators that evaluate perspectives directed at the mental health of these patients. In this scoping review, indications related to post-traumatic stress disorder were excluded because it is a complication identified later, after the hospitalisation phase.

Also, regarding complications, three indicators were found to evaluate the occurrence of infectious complications. Infections, in conjunction with dysfunction and/or multiple organ failure, are considered the main mortality ratio in burn patients. For burns above 20% of the body surface, in addition to the rupture of the protective skin barrier, humoral and cellular immunity are also altered, making preventing and treating infection more difficult [123]. A prospective cohort study conducted with adult burn patients admitted to an ICU observed a 26% prevalence of sepsis, overall mortality of 11.9%, and 34.4% in patients with sepsis [124], indicating the importance that this type of complication has in the final result obtained and, therefore, the need to monitor these events for better quality care.

Other complications mentioned in the articles were acute respiratory distress syndrome, venous thromboembolism and pulmonary embolism, cardiac arrest, and bacteremia, among others.

Despite the extensive list of process indicators found, some aspects were not considered, such as data related to speech therapy and speech/swallowing complications.

Regarding outcome indicators, the most cited was the "length of hospital stay". Initially, mortality was the only measure of the quality of hospital care adopted. As some standards of care have been established and practices have changed and improved, fortunately, the survivability of burns has increased significantly. Thus, the need to include new indicators arose [125]. In addition to the length of hospital stay, the inclusion of a greater number of physical-functional indicators can also be observed. The scarring changes, developed by excessive skin fibrosis, lead to joint contractures that are associated with changes in muscle strength and functional capacity due to long periods of sedation and immobility and the hypercatabolic state of these patients, leading to physical-functional changes that can impact the quality of life in the long term [72]. A retrospective study found, as the main finding, that extremities contracture is independently associated with a lower return to work at 6, 12, and 24 months after the injury [126]. New strategies implemented in acute care for these patients, such as excision and closure as soon as possible, early mobility strategies, lighter sedations, and previous resumption of exercises after grafting, can impact both the physical-functional results and the length of hospital stay [126], emphasising the importance of using these indicators to evaluate and monitor the care provided.

### Study limitations

In our study, we opted not to include grey literature due to the difficulty in retrieving the data and their low reliability because they have not been peer-reviewed. The inclusion of the grey literature could have led to the identification of some other aspect of care, but given the number of studies included, we believe that the inclusion would have added little or nothing to the final results. Another limitation of this study is that despite various efforts, four articles considered eligible by reading their titles and abstracts were not found in their entirety to assess eligibility after full reading. In addition, it was not possible to evaluate the methodological quality of all included studies, although scoping reviews are not required. It was chosen not to delimit the research about the study design in an attempt to encompass a greater number of hospital quality indicators.

Some indicators found in the studies were not included in the data collection, such as indicators related to the evaluation of scars, the cost of care, aspects related to pre-hospital care, adverse effects of a certain procedure that was being analysed in the study, long-term quality of life and post-traumatic stress, as they are related to aspects of care not included in the scope of this study.

## Conclusion

This scoping review was performed to identify quality indicators for hospital care of acute burn patients. It is part of a larger project that aims to build an instrument to evaluate the quality of care provided by Brazilian burn units. As a result of this study, a list of indicators already used was obtained, which will be further reviewed by a group of experts. In addition, this list may also be useful for further research or implementation in a program to improve the quality of hospital care provided to acute burn patients. The human resources needed to obtain better results and indicators related to speech therapy and speech/swallowing complications in burned patients were not covered.

### Supplementary Information

**Supplementary Material 1. **

**Supplementary Material 2. **

**Supplementary Material 3. **

**Supplementary Material 4. **

## Data Availability

All data generated or analysed during this study are included in this article and its supplemental information files.

## Abbreviations

WHO: World Health Organization
PRISMA-ScR: PRISMA Extensions for Scoping Reviews
BMI: Body mass index
TBSA: Total body surface area
VAP: Ventilation-Associated Pneumonia
ICU: Intensive Care Unit
LA50: Lethal Area 50 – Total Burned Body Surface with 50% mortality
ABA: American Burn Association
EBA: European Burns Association
ISBI: International Society for Burn Injuries
ARDS: Acute Respiratory Distress Syndrome
