# Supplementary material for: Quality indicators for hospital burn care: a scoping review
Source: BMC Health Serv Res. 2024 Apr 19;24:486. doi: 10.1186/s12913-024-10980-7 (PMC11031897; doi:10.1186/s12913-024-10980-7)
Supplement: Supplementary file 1 — Supplementary Material 1. [file 12913_2024_10980_MOESM1_ESM.docx]

**Additional file 1** – Search strategy for MEDLINE

| **#1** | (Burns[Title/Abstract]) OR (Burn[Title/Abstract]) |
| --- | --- |
| **#2** | (((Burns, Inhalation[Title/Abstract]) OR (Inhalation Burns[Title/Abstract])) OR (Burn, Inhalation[Title/Abstract])) OR (Inhalation Burn[Title/Abstract]) |
| **#3** | (((((Smoke Inhalation Injury[Title/Abstract]) OR (Inhalation Injury, Smoke[Title/Abstract])) OR (Injury, Smoke Inhalation[Title/Abstract])) OR (Inhalation Injuries, Smoke[Title/Abstract])) OR (Injuries, Smoke Inhalation[Title/Abstract])) OR (Smoke Inhalation Injuries[Title/Abstract]) |
| **#4** | (((Burns, Electric[Title/Abstract]) OR (Electric Burns[Title/Abstract])) OR (Burn, Electric[Title/Abstract])) OR (Electric Burn[Title/Abstract]) |
| **#5** | (((Burns, Chemical[Title/Abstract]) OR (Chemical Burns[Title/Abstract])) OR (Burn, Chemical[Title/Abstract])) OR (Chemical Burn[Title/Abstract]) |
| **#6** | #1 OR #2 OR #3 OR #4 OR #5 |
| **#7** | (((((((((((((((Quality Assurance, Health Care[Title/Abstract]) OR (Healthcare Quality Assurance[Title/Abstract])) OR (Assurance, Healthcare Quality[Title/Abstract])) OR (Assurances, Healthcare Quality[Title/Abstract])) OR (Healthcare Quality Assurances[Title/Abstract])) OR (Quality Assurances, Healthcare[Title/Abstract])) OR (Quality Assurance, Healthcare[Title/Abstract])) OR (Health Care Quality Assurance[Title/Abstract])) OR (Healthcare Quality Assessment[Title/Abstract])) OR (Assessment, Healthcare Quality[Title/Abstract])) OR (Assessments, Healthcare Quality[Title/Abstract])) OR (Healthcare Quality Assessments[Title/Abstract])) OR (Quality Assessments, Healthcare[Title/Abstract])) OR (Quality Assessment, Healthcare[Title/Abstract])) OR (Quality Assessment, Health Care[Title/Abstract])) OR (Health Care Quality Assessment[Title/Abstract]) |
| **#8** | (((Quality Improvement[Title/Abstract]) OR (Improvement, Quality[Title/Abstract])) OR (Improvements, Quality[Title/Abstract])) OR (Quality Improvements[Title/Abstract]) |
| **#9** | (((((((((((Quality Indicators, Health Care[Title/Abstract]) OR (Quality Indicators, Healthcare[Title/Abstract])) OR (Healthcare Quality Indicator[Title/Abstract])) OR (Healthcare Quality Indicators[Title/Abstract])) OR (Indicator, Healthcare Quality[Title/Abstract])) OR (Indicators, Healthcare Quality[Title/Abstract])) OR (Quality Indicator, Healthcare[Title/Abstract])) OR (Health Metrics[Title/Abstract])) OR (Health Metric[Title/Abstract])) OR (Metrics, Health[Title/Abstract])) OR (Global Trigger Tool, Healthcare[Title/Abstract])) OR (Healthcare Global Trigger Tool[Title/Abstract]) |
| **#10** | (Health Care Quality, Access and Evaluation[Title/Abstract]) OR (Healthcare Quality, Access and Evaluation[Title/Abstract]) |
| **#11** | ((((((Health Care Evaluation Mechanisms[Title/Abstract]) OR (Healthcare Evaluation Mechanisms[Title/Abstract])) OR (Evaluation Mechanism, Healthcare[Title/Abstract])) OR (Evaluation Mechanisms, Healthcare[Title/Abstract])) OR (Healthcare Evaluation Mechanism[Title/Abstract])) OR (Mechanism, Healthcare Evaluation[Title/Abstract])) OR (Mechanisms, Healthcare Evaluation[Title/Abstract]) |
| **#12** | (((((((Patient Reported Outcome Measures[Title/Abstract]) OR (Patient Reported Outcome Measure[Title/Abstract])) OR (Patient Reported Outcomes[Title/Abstract])) OR (Outcome, Patient Reported[Title/Abstract])) OR (Patient Reported Outcome[Title/Abstract])) OR (Patient-Reported Outcome[Title/Abstract])) OR (Outcome, Patient-Reported[Title/Abstract])) OR (Patient-Reported Outcomes[Title/Abstract]) |
| **#13** | (((((((Outcome and Process Assessment, Health Care[Title/Abstract]) OR (Outcome and Process Assessment[Title/Abstract])) OR (Outcome and Process Assessment, Health Care[Title/Abstract]))) OR (Structure Process Outcome Triad[Title/Abstract])) OR (Donabedian Model[Title/Abstract])) OR (Model, Donabedian[Title/Abstract])) OR (Donabedian Triad[Title/Abstract])) OR (Triad, Donabedian[Title/Abstract]) |
| **#14** | ((((((((Quality of Health Care[Title/Abstract]) OR (Health Care Quality[Title/Abstract])) OR (Quality of Healthcare[Title/Abstract])) OR (Healthcare Quality[Title/Abstract])) OR (Quality of Care[Title/Abstract])) OR (Care Quality[Title/Abstract])) OR (Pharmacy Audit[Title/Abstract])) OR (Audit, Pharmacy[Title/Abstract])) OR (Pharmacy Audits[Title/Abstract]) |
| **#15** | #7 OR #8 OR #9 OR #10 OR #11 OR #12 OR #13 OR #14 |
| **#16** | #6 AND #15 |
