# Supplementary material for: Quality indicators for hospital burn care: a scoping review
Source: BMC Health Serv Res. 2024 Apr 19;24:486. doi: 10.1186/s12913-024-10980-7 (PMC11031897; doi:10.1186/s12913-024-10980-7)
Supplement: Supplementary file 2 — Supplementary Material 2. [file 12913_2024_10980_MOESM2_ESM.docx]

**Additional file 2 –** Developed form for data extraction

| Title |  |
| --- | --- |
| Authors |  |
| Journal / Year of publication |  |
| Language of publication |  |
| Country |  |
| Study design |  |
| Quality indicator | ( ) Structure ( ) Process ( ) Outcome |
| Purpose of the indicator |  |
| Formula indicator |  |
| Eligibility | ( ) Include ( ) Exclude – Reason: |
