# Supplementary material for: Quality indicators for hospital burn care: a scoping review
Source: BMC Health Serv Res. 2024 Apr 19;24:486. doi: 10.1186/s12913-024-10980-7 (PMC11031897; doi:10.1186/s12913-024-10980-7)
Supplement: Supplementary file 3 — Supplementary Material 3. [file 12913_2024_10980_MOESM3_ESM.docx]

**Additional file 3 –** Characteristics of the included studies

| SYSTEMATIC REVIEWS | | | | | | |
| --- | --- | --- | --- | --- | --- | --- |
| Title | Year | Journal | Language | Country | AMSTAR 2 | Quality Indicators |
| Predicting length of stay in thermal burns: A systematic review of prognostic factors^94^ | 2013 | Burns | English | United Kingdom | Critically low | - length of hospital stay |
| Oxandrolone use in adult burn patients. Systematic review and meta-analysis^57^ | 2014 | Acta Cirurgica Brasileira | English | Brazil | Critically low | - healing time of the donor area  - laboratory diet markers  - weight loss during hospitalisation  - length of hospital stay  - monitoring of hypermetabolism |
| Topical treatment for facial burns^35^ | 2020 | Cochrane Library | English | The Netherlands | Moderate | - time to wound closure/percentage of wound healed  - care-associated infection  - pain assessment  - length of hospital stay  - patient satisfaction  - need for surgery |
| Pediatric enteral nutrition therapy for burn victims: when should it be initiated?^55^ | 2019 | Rev. Brasileira de Terapia Intensiva | Portuguese | Brazil | Critically low | - mortality  - length of hospital stay  - energy and protein needs/consumption  - weight loss during hospitalisation  - diet-related complications  - laboratory diet markers |
| Tissue healing efficacy in burn patients treated with 1% silver sulfadiazine versus other treatments: a systematic review and meta-analysis of randomized controlled trials^100^ | 2019 | Anais Brasileiros de Dermatologia | English | Brazil | Low | - time to wound closure |
| Meta-analysis of early excision of burns^62^ | 2006 | Burns | English | Singapore | Critically low | - mortality  - assessment of blood loss  - length of hospital stay  - duration of sepsis  - duration of surgical procedures  - number of surgeries  - time to wound closure |

| RANDOMISED CONTROLLED TRIALS | | | | | | | | | | | | | | | | |
| --- | --- | --- | --- | --- | --- | --- | --- | --- | --- | --- | --- | --- | --- | --- | --- | --- |
| Title | Year | Journal | Language | Country | PEDro Scale | | | | | | | | | | | Quality Indicators |
| Prospective randomized controlled trial comparing the effects of noncontact low frequency ultrasound with standard care in healing split-thickness donor sites^33^ | 2015 | Journal of the American College of Surgeons | English | USA | 1  Y | 2  Y | 3  Y | 4  Y | 5  N | 6  N | 7  Y | 8  N | 9  Y | 10  Y | 11  Y | - healing time  - assessment of pain/pruritus  - reopening rate of the donor area |
| Evaluation of Liposomal Bupivacaine at split-thickness skin graft donor sites through a randomized, controlled trial^34^ | 2021 | Journal of Burn Care & Research | English | USA | 1  Y | 2  Y | 3  N | 4  Y | 5  Y | 6  N | 7  Y | 8  N | 9  Y | 10  Y | 11  Y | - pain assessment  - time of first opioid medication  - registration of pain medications |
| The effect of reflexology massage and passive music therapy intervention before burn dressing on pain, anxiety level and sleep quality^36^ | 2022 | Burns | English | Turkey | 1  Y | 2  Y | 3  N | 4  Y | 5  N | 6  N | 7  N | 8  N | 9  Y | 10  Y | 11  Y | - pain/anxiety assessment  - sleep quality assessment  - use of opioids and analgesics |
| Rapid enzymatic burn debridement: A review of the paediatric clinical trial experience^44^ | 2020 | International Wound Journal | English | Israel / Germany | 1  Y | 2  Y | 3  N | 4  N | 5  N | 6  N | 7  N | 8  N | 9  Y | 10  Y | 11  Y | - time to wound closure  - time to complete removal of the eschar  - blood loss  - percentage of wound grafted  - incidence and excised wound area  - need for escharotomy |
| Comparative study of conventional and topical heparin treatments for burns analgesia^70^ | 2010 | Rev. Associação Médica Brasileira | Portuguese | Brazil | 1  Y | 2  Y | 3  N | 4  N | 5  N | 6  N | 7  N | 8  N | 9  Y | 10  Y | 11  Y | - demand for analgesics |
| Cost-effectiveness of collagen wound dressing with alginate for the treatment of split-thickness skin graft donor sites ^38^ | 2017 | Rev. Brasileira de Queimaduras | Portuguese | Brazil | 1  Y | 2  Y | 3  N | 4  N | 5  N | 6  N | 7  N | 8  N | 9  Y | 10  Y | 11  Y | - pain assessment  - length of hospital stay  - time for epithelialisation |
| Randomized controlled trial of the foot reflexology on pain and anxiety severity during dressing change in burn patients^39^ | 2021 | Burns | English | Iran | 1  Y | 2  Y | 3  N | 4  Y | 5  N | 6  N | 7  Y | 8  N | 9  Y | 10  Y | 11  Y | - pain/anxiety assessment |
| The effects of short bouts of ergometric exercise for severely burned children in intensive care: A randomized controlled trial^98^ | 2022 | Clinical Rehabilitation | English | USA | 1  Y | 2  Y | 3  Y | 4  Y | 5  N | 6  N | 7  Y | 8  N | 8  Y | 10  Y | 11  Y | - length of ICU hospitalisation  - body mass index  - deambulation |
| Controlling intraoperative hemorrhage during burn surgery: A prospective, randomized trial comparing NuStat1 hemostatic dressing to thehistoric standard of care^83^ | 2017 | Burns | English | USA | 1  Y | 2  Y | 3  N | 4  Y | 5  N | 6  N | 7  N | 8  N | 9  Y | 10  Y | 11  Y | - blood loss |
| Gauze dressing versus cellulose fiber salinized mesh as a temporary dressing for skin graft donor sites^41^ | 2015 | Revista Brasileira de Queimaduras | Portuguese | Brazil | 1  Y | 2  Y | 3  N | 4  N | 5  N | 6  N | 7  N | 8  N | 9  Y | 10  Y | 11  Y | - pain assessment  - re-epithelialisation of the donor area  - wound complications |
| The effect of progressive muscle relaxation on anxiety and sleep quality in burn patients: A randomized clinical trial^86^ | 2019 | Burns | English | Iran | 1  Y | 2  Y | 3  Y | 4  Y | 5  N | 6  N | 7  N | 8  Y | 9  Y | 10  Y | 11  Y | - anxiety and sleep quality assessment |
| A novel rapid and selective enzymatic debridement agent for burn wound management: A multi-center RCT^45^ | 2014 | Burns | English | USA/ Israel | 1  Y | 2  Y | 3  Y | 4  Y | 5  N | 6  N | 7  N | 8  Y | 9  Y | 10  Y | 11  Y | - quantity and percentage of wound excised and grafted  - time to complete removal of the eschar  - time to wound closure  - need for escharotomy  - blood loss |
| Hypnosis for reduction of background pain and pain anxiety in men with burns: A blinded, randomised, placebo-controlled study^42^ | 2017 | Burns | English | Iran | 1  Y | 2  Y | 3  N | 4  Y | 5  Y | 6  N | 7  Y | 8  Y | 9  Y | 10  Y | 11  Y | - assessment of pain intensity  - assessment of pain quality  - anxiety |
| Computer tablet distraction reduces pain and anxiety in pediatric burn patients undergoing hydrotherapy: A randomized trial^43^ | 2017 | Burns | English | USA | 1  Y | 2  Y | 3  Y | 4  Y | 5  N | 6  N | 7  N | 8  Y | 9  Y | 10  Y | 11  Y | - pain assessment  - emotional manifestation of pain  - nursing perception of the pain  - duration of hydrotherapy |

| QUASI-EXPERIMENTAL PROSPECTIVE STUDIES | | | | | | | | | | | | | | | | |
| --- | --- | --- | --- | --- | --- | --- | --- | --- | --- | --- | --- | --- | --- | --- | --- | --- |
| Title | Year | Journal | Language | Country | PEDro Scale | | | | | | | | | | | Quality Indicators |
| A quality improvement project incorporating preoperative warming to prevent perioperative hypothermia in major burns.^81^ | 2018 | Burns | English | Canada | 1  Y | 2  N | 3  N | 4  Y | 5  N | 6  N | 7  N | 8  N | 9  Y | 10  Y | 11  Y | - perioperative hypothermia |
| The use of a Bioactive skin substitute decreases length of stay for pediatric burn patients^69^ | 2001 | Journal of Pediatric Surgery | English | USA | 1  Y | 2  N | 3  N | 4  Y | 5  N | 6  N | 7  N | 8  Y | 9  Y | 10  Y | 11  Y | - length of hospital stay  - need for autografting |
| Volume-based feeding improves nutritional adequacy in surgical patients^56^ | 2018 | The American Journal of Surgery | English | USA | 1  Y | 2  N | 3  N | 4  Y | 5  N | 6  N | 7  N | 8  N | 9  Y | 10  Y | 11  Y | - percentage of target protein and calories delivered/volume delivered per probe  - length of hospital stay  - mortality  - surgical site infection/pneumonia  - need pro-kinetic/gastrointestinal intolerance/emesis  - bronchoalveolar aspiration |
| Investigating the effect of seeing patients’ pre-burn facephoto on the quality of care and level of empathy of nurses with patients admitted to BICU^88^ | 2021 | Burns | English | Iran | 1  Y | 2  N | 3  N | 4  N | 5  N | 6  N | 7  N | 8  N | 9  Y | 10  Y | 11  Y | - nurse empathy with patient  - patient satisfaction/caregiver with the quality of nursing care |
| Effect of nitrous oxide on fentanyl consumption in burned patients undergoing dressing change^37^ | 2016 | Rev. Brasileira de Anestesiologia | Portuguese | Brazil | 1  Y | 2  N | 3  N | 4  N | 5  N | 6  N | 7  N | 8  N | 9  Y | 10  Y | 11  Y | - drugs for analgesic control  - dose of morphine and time between doses  - pain assessment  - intravenous fentanyl demand  - patient satisfaction |
| Physiotherapeutic intervention in burned patient: a respiratory therapy approach in a pilot study^104^ | 2016 | Rev. Brasileira de Queimaduras | Portuguese | Brazil | 1  Y | 2  N | 3  N | 4  N | 5  N | 6  N | 7  N | 8  N | 9  Y | 10  Y | 11  Y | - respiratory therapy results |
| Hospital-acquired infections and thermally injured patients: Chlorhexidine gluconate baths work^75^ | 2014 | American Journal of Infection Control | English | USA | 1  N | 2  N | 3  N | 4  Y | 5  N | 6  N | 7  N | 8  N | 9  Y | 10  Y | 11  Y | - hospital-acquired infections |
| Improving hand hygiene in a low-resource setting: A nurse-led quality improvement Project^15^ | 2021 | International Wound Journal | English | Malawi | 1  N | 2  N | 3  N | 4  N | 5  N | 6  N | 7  N | 8  N | 9  Y | 10  Y | 11  Y | - incidence of hand hygiene  - structure for hand hygiene |
| A performance improvement initiative to determine the impact of increasing the time interval between changing centrally placed intravascular catheters^76^ | 2014 | Journal of Burn Care & Research | English | USA | 1  Y | 2  N | 3  N | 4  Y | 5  N | 6  N | 7  N | 8  N | 9  Y | 10  Y | 11  Y | - central catheter infection rate |
| Mobilization practices for patients with burn injury in critical care^72^ | 2020 | Burns | English | Brazil | 1  Y | 2  N | 3  N | 4  N | 5  N | 6  N | 7  N | 8  N | 9  Y | 10  Y | 11  Y | - muscle strength assessment  - mobility level  - deambulation |

| PROSPECTIVE COHORT STUDIES | | | | | | | | |
| --- | --- | --- | --- | --- | --- | --- | --- | --- |
| Title | Year | Journal | Country | Language | Newcastle-Ottawa | | | Quality Indicators |
| [Practice of nosocomial infection management in burn department based on the American hospital evaluation standard of the Joint Commission International].^73^ | 2020 | Chinese Journal of Burns | Mandarin | China | Selection | Comparability  ** | Outcome  * | - hand hygiene rate  - hand hygiene accuracy rate  - infection of central venous catheter/urinary tract/VAP  - prevention of catheter infections  - specification of catheter fixation  - preventive measures of VAP |
| Which of the Abbreviated Burn Severity Index variables are having impact on the hospital length of stay?^23^ | 2007 | Journal of Burn Care & Research | English | Austria | Selection  ** | Comparability  ** | Outcome  *** | - prediction of mortality  - length of hospital stay |
| Benchmarking outcomes in the critically injured burn patient^77^ | 2014 | Annals of Surgery Open | English | USA | Selection  ** | Comparability  ** | Outcome  ** | - organ dysfunction  - infectious complications  - mortality  - time to recover organ dysfunction |
| Food profile of a specialized public hospital for patients injured by burns^32^ | 2017 | Revista Brasileira de Queimaduras | Portuguese | Brazil | Selection  ** | Comparability  * | Outcome  ** | - energy and protein needs  - quantity of calories and proteins offered and consumed  - calculation of body mass index  - percentage of diet acceptance |
| Early excision and grafting versus delayed excision and grafting of deep termal burns up to 40% total body surface área: A comparison of outcome.^78^ | 2012 | Annals of Burns and Fire Disasters | English | Pakistan | Selection  *** | Comparability  ** | Outcome  ** | - wound with positive culture  - graft setting percentage  - length of hospital stay  - need for blood transfusion |
| Early enteral nutrition in burns: compliance with guidelines and associated outcomes in a multicenter study^53^ | 2011 | Journal of Burn Care & Research | English | USA | Selection  *** | Comparability  ** | Outcome  *** | - initiation of enteral nutrition  - organ dysfunction  - infectious complications  - days of enteral nutrition  - length of ICU hospitalisation  - mortality |

| RETROSPECTIVE COHORT STUDIES | | | | | | | | | | |
| --- | --- | --- | --- | --- | --- | --- | --- | --- | --- | --- |
| Title | Year | Journal | Language | Country | Newcastle-Ottawa | | | | Quality Indicators |  |
| A predictive model for prolonged hospital length of stay in surgical burn patients^92^ | 2020 | Burns | English | USA | Selection  ** | Comparability | | Outcome  *** | - length of hospital stay |  |
| Analysis of factors impacting length of stay in thermal and inhalation injury^93^ | 2019 | Burns | English | USA | Selection  ** | Comparability | | Outcome  *** | - length of hospital stay |  |
| Contributors to the length-of-stay trajectory in burn-injured patients^63^ | 2018 | Burns | English | Canada | Selection  ** | Comparability  ** | | Outcome  ** | - length of hospital stay/total body surface area  - hospital complications  - days of enteral nutrition  - surgical procedures |  |
| Early enteral nutrition in geriatric burn patients: Is there a benefit?^52^ | 2020 | Journal of Burn Care & Research | English | USA | Selection  *** | Comparability  ** | | Outcome  ** | - initiation of enteral nutrition  - length of hospital stay/ICU  - hospital complications  - mortality |  |
| LA50 in burns injuries^91^ | 2016 | Annals of Burns and Fire Disasters | English | Iran | Selection  ** | Comparability | | Outcome  ** | - mortality: LA50 |  |
| Accuracy of commercial reporting systems to monitor quality of care in burns^22^ | 2014 | Burns | English | United Kingdom | Selection  *** | Comparability  * | | Outcome  ** | - prediction of mortality |  |
| Not all patients meet the 1day per percent burn rule: A simple method for predicting hospital length of stay in patients with burn^95^ | 2016 | Burns | English | USA | Selection  * | Comparability  ** | | Outcome  * | - length of hospital stay |  |
| Patient safety measures in burn care: Do national reporting systems accurately reflect quality of burn care?^87^ | 2010 | Journal of Burn Care & Research | English | USA | Selection  ** | Comparability  ** | | Outcome  ** | - expected mortality  - complications |  |
| Length of stay per total body surface area burn relative to mechanism: A pediatric injury quality improvement collaborative study^99^ | 2022 | Journal of Burn Care & Research | English | USA | Selection  * | Comparability  ** | | Outcome  ** | - length of hospital stay/total body surface area |  |
| Epidemiology of burn at a military hospital in Bahrain: initial experience of patient outcomes and quality indicators^90^ | 2018 | International Journal of Burns and Trauma | English | Saudi Arabia | Selection  ** | Comparability | | Outcome  ** | - length of hospital stay/ICU  - mortality |  |
| Unplanned readmission after traumatic injury: A long-term nationwide analysis^110^ | 2019 | Journal Trauma Acute Care Surgery | English | USA | Selection  ** | Comparability  ** | | Outcome  ** | - readmission at 1, 3, and 6 months/total hospitalised patients |  |
| Prolonged hospital length of stay in pediatric trauma: a model for targeted interventions^96^ | 2020 | Pediatric Research | English | USA | Selection  ** | Comparability | | Outcome  ** | - length of hospital stay |  |
| Evaluation of burn care utilizing a National Burn Registry^16^ | 1978 | Emergency Medical Services | English | USA | Selection  * | Comparability  ** | | Outcome  * | - expected mortality  - number of grafts  - anaesthesia time  - days from first to last grafting  - days from injury to last grafting  - length of hospital stay  - resuscitation – fluid volume/urinary output  - burned region  - conformity with infection control procedures  - existence and implementation of a care plan  - involvement with teaching  - admission of overload patient  - existence of admission protocol |  |
| Mortalidad tras un año de protocolización en el manejo del paciente quemado^65^ | 2010 | Revista Chilena de Cirurgia | Spanish | Chile | Selection  * | Comparability | | Outcome  ** | - days of enteral nutrition  - length of hospital stay/ICU  - duration of surgical procedures  - expected mortality |  |
| Factor XIII-guided treatment algorithm reduces blood transfusion in burn surgery^82^ | 2018 | Rev. Brasileira de Anestesiologia | Portuguese | Portugal | Selection  *** | Comparability  * | | Outcome  ** | - quantity of blood product usage in the perioperative period  - coagulation biomarkers |  |
| Contracture severity at hospital discharge in children: A burn model system database study^101^ | 2021 | Journal of Burn Care & Research | English | USA | Selection  ** | Comparability | | Outcome  *** | - active ROM at discharge |  |
| Length of stay per total body surface area burn: A validation study using the national burn registry^27^ | 2022 | Journal of Burn Care & Research | English | USA | Selection  ** | Comparability | | Outcome  *** | - length of hospital stay/total body surface area  - prediction of mortality  - mortality |  |
| Mortality analysis of adult burn patients in Uruguay^17^ | 2020 | Rev. Brasileira de Terapia Intensiva | Portuguese | Uruguay | Selection  *** | Comparability  ** | | Outcome  *** | - expected mortality  - gross mortality  - LA50  - presence of inhalation injury  - length of ICU hospitalisation  - days of enteral nutrition |  |
| Establishing a hospital transfusion management system promotes appropriate clinical use of human albumin in Japan: a nationwide retrospective study^49^ | 2019 | BMC Health Services Research | English | Japan | Selection  ** | Comparability  ** | | Outcome  ** | - gross mortality  - length of hospital stay  - albumin use |  |
| Modified first world mortality scores can be used in a regional South African burn service with resource limitations.^25^ | 2016 | Burns | English | South Africa | Selection  ** | Comparability  ** | | Outcome  ** | - prediction of mortality |  |
| Systems-based practice in burn care prevention, management, and economic impact of health care–associated infections^18^ | 2017 | Clinics in Plastic Surgery | English | USA | Selection  ** | Comparability  ** | | Outcome  ** | - care-associated infections  - identification of pathogens  - presence of inhalation injury  - length of hospital stay  - mortality |  |
| Hospital-acquired pressure ulcer prevention: A burn surgeon’s team approach^80^ | 2014 | Journal of Burn Care & Research | English | USA | Selection  * | Comparability | | Outcome  ** | - incidence of decubitus ulcers |  |
| Comparison of two healing techniques in pediatric burned patients^59^ | 2018 | Revista Pediatría Eletrónica | Spanish | Chile | Selection  ** | | Comparability  ** | Outcome  ** | - quantity that needed grafting  - grafting percentage  - length of hospital stay  - number of dressings at admission  - days from injury to grafting  - infectious complications |  |
| Comparison of severity scores for mortality prediction and length of hospital stay in burn units^26^ | 2017 | Revista Brasileira de Queimaduras | Portuguese | Brazil | Selection  *** | | Comparability  ** | Outcome  ** | - prediction of mortality  - length of hospital stay  - mortality |  |
| Use of albumin as a risk factor for hospital mortality among burn patients in Brazil: non-concurrent cohort study^50^ | 2010 | São Paulo Medical Journal | English | Brazil | Selection  *** | | Comparability  ** | Outcome  ** | - mortality  - length of hospital stay  - albumin use |  |
| Effectiveness of an Early Mobilization Protocol in a Trauma and Burns Intensive Care Unit: A Retrospective Cohort Study^84^ | 2013 | Physical Therapy | English | USA | Selection  ** | | Comparability  ** | Outcome  ** | - mobilisation adverse events  - length of hospital stay  - days of enteral nutrition  - mortality |  |
| Effects of mobility training on severe burn patients in the BICU: A retrospective cohort study^71^ | 2016 | Burns | English | China | Selection  ** | | Comparability  ** | Outcome  *** | - range of motion  - mobility at discharge  - length of hospital stay/ICU  - duration of rehabilitation in the ICU  - days of enteral nutrition  - absolute bed rest time |  |
| Is the length of time in acute burn surgery associated with poorer outcomes?^68^ | 2014 | Burns | English | Australia | Selection  ** | | Comparability  ** | Outcome  *** | - duration of surgical procedures  - length of hospital stay  - infectious complication rate  - use of blood product usage  - graft loss |  |
| The impact of operative time and hypothermia in acute burn surgery66 | 2017 | Burns | English | Canada | Selection  ** | | Comparability  ** | Outcome  *** | - total duration of surgical procedures  - perioperative hypothermia  - postoperative complications |  |
| Abdominal compartment syndrome in the severely burned patient^48^ | 2007 | Journal of Burn Care & Research | English | USA | Selection  ** | | Comparability  * | Outcome  *** | - intra-abdominal hypertension  - urinary output  - organ failure  - Ivy score |  |
| Adult contractures in burn injury: A burn model system national satabase study^102^ | 2017 | Journal of Burn Care & Research | English | USA | Selection  *** | | Comparability  * | Outcome  *** | - number of joint contractures  - ectropion, microstomia, and nasolabial contractures |  |
| Quantifying contracture severity at hospital discharge in adults: A burn model system national database study^103^ | 2018 | Journal of Burn Care & Research | English | USA/  Canada | Selection  ** | | Comparability  * | Outcome  *** | - active ROM at discharge |  |
| A comparison of Biobrane™ and cadaveric allograft for temporizing the acute burn wound: Cost and procedural time^67^ | 2015 | Burns | English | Canada | Selection  *** | | Comparability  * | Outcome  *** | - duration of surgical procedures  - need for another debridement |  |

| CASE-CONTROL STUDIES | | | | | | | | |
| --- | --- | --- | --- | --- | --- | --- | --- | --- |
| Title | Year | Journal | Language | Country | Newcastle-Ottawa | | | Quality Indicators |
| Implementation of a nurse-driven burn resuscitation protocol: A quality improvement project^46^ | 2013 | Critical care nurse | English | USA | Selection  ** | Comparability  ** | Exposure | - indication of resuscitation fluid volume  - resuscitation fluid volume  - monitoring of resuscitation volume  - complications of resuscitation volume  - days of enteral nutrition  - length of hospital stay/ICU |
| Current status of nutritional care provision to burnt patients. Processes audit of a burnt patients department from a tertiary hospital^30^ | 2008 | Nutricion Hospitalaria | Spanish | Cuba | Selection  *** | Comparability  * | Exposure  * | - gross mortality  - complications  - length of hospital stay/total body surface area  - weight and height assessment at admission  - patients weighed at least once at hospitalisation  - value of serum albumin  - lymphocyte count  - patients with nutritional assessment  - patients with oral supplementation  - patients with indication of artificial nutritional support  - patients with sufficient energy intake  - patients with nutritional balance |
| A comparative analysis from the morbidity and mortality before and after the deployment burn protocol^60^ | 2014 | Rev. Brasileira de Queimaduras | Portuguese | Brazil | Selection  ** | Comparability  ** | Exposure  * | - length of hospital stay  - transfers  - gross mortality  - surgical procedures  - number of grafts |
| Pediatric burn-trauma patients have increased length of stay compared to trauma-only patients: A propensity matched analysis^74^ | 2020 | Burns | English | USA | Selection  ** | Comparability  ** | Exposure  ** | - length of hospital stay  - mortality  - days of enteral nutrition  - infection, sepsis, and other complications |
| Colloid administration normalizes resuscitation ratio and ameliorates “fluid creep”^47^ | 2010 | Journal of Burn Care & Research | English | USA | Selection  ** | Comparability  * | Exposure  *** | - resuscitation – fluid volume/urinary output |
| Impact of a computerized information system on quality of nutritional support in the ICU^28^ | 2006 | Nutrition | English | Switzerland | Selection  ** | Comparability  ** | Exposure  * | - start of feeding  - energy target and computed intake  - energy target vs. energy supplied  - weight loss  - patient weight at admission  - artificial feeding |
| Use of Transcyte and dermabrasion to treat burns reduces length of stay in burns of all size and etiology^97^ | 2006 | Burns | English | USA | Selection  *** | Comparability  ** | Exposure | - length of hospital stay |
| The use of a novel burn dressing out of bacterial nanocellulose compared to the French standard of care in paediatric 2nd degree burns – A retrospective analysis^40^ | 2022 | Burns | English | France | Selection  ** | Comparability  ** | Exposure  ** | - wound healed on the fifteenth day  - time for complete healing  - need for grafting  - length of hospital stay  - pain assessment  - wound infection  - patient and/or family satisfaction |
| Early fluid resuscitation improves outcomes in severely burned children^19^ | 2000 | Resuscitation | English | USA | Selection  ** | Comparability  ** | Exposure  *** | - time from injury to resuscitation  - time until first surgical excision  - incidence of sepsis  - incidence of renal failure/CRP  - presence of inhalation injury  - mortality |

| CROSS-SECTIONAL STUDIES | | | | | | | | | |
| --- | --- | --- | --- | --- | --- | --- | --- | --- | --- |
| Title | Year | Journal | Language | Country | Newcastle-Ottawa | | | Quality Indicators |  |
| Effectiveness of the Modified Brooke Formula of Hydric Substitution for Management of the Burnt Patient^51^ | 2019 | Revista Cubana de Medicina General Integral | Spanish | Cuba | Selection  * | Comparability  ** | Outcome  * | - monitoring of resuscitation volume |  |
| Quality of the hospital care to children with lesions due to burns^24^ | 2011 | Medisan | Spanish | Cuba | Selection  * | Comparability  ** | Outcome  * | - prediction of mortality  - expected mortality |  |
| Patient satisfaction in a Spanish burn unit^108^ | 2018 | Burns | English | Spain | Selection  * | Comparability  ** | Outcome  ** | - patient satisfaction |  |
| Quality of life of patients in a burned treatment unit^105^ | 2011 | Rev. Brasileira de Cirurgia Plástica | Portuguese | Brazil | Selection  * | Comparability  ** | Outcome  * | - quality of life |  |
| Analysis of the burned patients' life quality submitted to the handling physical therapist interned in the Center of Handling of Burned^106^ | 2013 | Rev. Brasileira de Queimaduras | Portuguese | Brazil | Selection  ** | Comparability  ** | Outcome | - quality of life |  |
| Quality of life of burned patients in a referral hospital in northeastern Brazil ^107^ | 2013 | Rev. Brasileira de Queimaduras | Portuguese | Brazil | Selection  ** | Comparability  ** | Outcome  * | - quality of life |  |
| Patient reported experiences at a swedish national burn centre^109^ | 2022 | Journal of Burn Care & Research | English | Sweden | Selection  *** | Comparability  ** | Outcome  * | - patient satisfaction |  |
| Burn-related factors affecting anciety, depression and self-esteem in burn patients: na exploratory study^85^ | 2017 | Annals of Burns and Fire Disasters | English | India | Selection  * | Comparability  ** | Outcome  ** | - anxiety, depression, and self-esteem assessment |  |

| EXPERT CONSENSUS | | | | | | |
| --- | --- | --- | --- | --- | --- | --- |
| Title | Year | Journal | Language | Country | Quality Indicators |  |
| Developing clinical quality indicators for a Bi-National Burn Registry^5^ | 2011 | Burns | English | Australia/ New Zealand | - presence of a surgeon 24/7 and surgeon evaluation/nurse within 24 hours of admission  - multidisciplinary team weekly meetings  - routine swabs at admission/surveillance of infection at admission  - physical-functional assessment within 48 hours of admission  - patient weighed within 3–5 days of admission /weight loss at admission  - date and time of first surgical excision and time for complete excision of deep injuries  - initiation of enteral/parenteral nutrition  - negative change of GFR (eGFR)  - unplanned readmission  - length of hospital stay/ICU  - mechanical ventilator time  - gross mortality |  |
| Driving improved burns care and patient outcomes through clinical registry data: A review of quality indicators in the Burns Registry of Australia and New Zealand^2^ | 2020 | Burns | English | Australia/ New Zealand | - physical-functional assessment within 48 hours of admission  - time for complete excision of deep injury  - initiation of enteral/parenteral nutrition  - hospital readmission/ICU  - palliative care management  - infectious surveillance  - malnutrition risk assessment  - pain assessment  - psychosocial assessment  - calculation of resuscitation volume  - anticoagulation prophylaxis  - escharotomy  - standardised diagram for calculating total body surface area |  |
| The development of outcome statements for burn care^7^ | 1997 | Semlnars In Perioperative Nursing | English | USA | - pre-and postoperative evaluation  - graft grip > 90% /donor area healing  - cardiac and circulatory complications – complications of resuscitation volume  - venous or arterial complications related to the central catheter  - pruritus, anxiety, and sleep quality assessment  - pain management  - presence of infection – wound sepsis, presence of respiratory and urinary tract infection  - metabolic balance  - ROM, muscle strength, mobility and deambulation at discharge  - nutritional status – need/intake and weight at hospital discharge  - pulmonary and AV status and pulmonary and AV recovery after inhalation  - wound healing and integrity of the unburned skin  - understanding of post-hospital care |  |
| Defining Benchmarks in Pediatric Burn Care: Inception of the Pediatric Injury Quality Improvement Collaborative^8^ | 2021 | Journal of Burn Care & Research | English | USA | - time until first surgical excision  - initiation of enteral nutrition  - psychological evaluation  - quantity of patients who needed grafting |  |
| Summary of the 2012 ABA burn quality consensus conference^9^ | 2013 | Journal of Burn Care & Research | English | USA | - depressive symptoms / acute stress disorder – initial assessment and follow up  - use of protocol for resuscitation volume and monitoring  - instruments for calculating total body surface area  - presence of inhalation injury/ need for intubation  - initiation of enteral nutrition  - need for decompression procedure  - expected mortality  - total protein and calorie intake/glutamine use/oxandrolone use  - weight loss  - mean weekly glucose and HR levels – diet markers  - physical-functional assessment in 48 hours  - quality of life  - time to complete removal of the eschar  - time to wound closure or percentage of open wound at discharge  - wound infection  - need for regrafting |  |

| CASE SERIES | | | | | | |
| --- | --- | --- | --- | --- | --- | --- |
| Title | Year | Journal | Language | Country | Quality Indicators |  |
| Synergistic use of novel technological advances in burn care significantly reduces hospital length of stay below predicted: A case series^61^ | 2022 | Journal of Burn Care & Research | English | USA | - length of hospital stay  - quantity of surgical procedures  - total duration of surgical procedures  - distance that patient can walk at discharge |  |
| RResults of the implementation of a nutritional support protocol for major burn pediatric patients hospitalized in the Intensive Care Unit^29^ | 2018 | Arquivos Argentinos de Pediatria | Spanish | Argentina | - time to start enteral nutritional and intake vs. weekly energy and protein target  - gross mortality  - weight assessment and BMI at admission  - basal metabolic rate  - nitrogen balance and laboratory diet markers |  |
| Effects of enteral nutritional therapy in burned patients treated at a public hospital in Joinville/SC^31^ | 2009 | Revista Brasileira de Queimaduras | Portuguese | Brazil | - BMI assessment  - biochemical parameters of nutrition  - registration of food consumption |  |

| INTEGRATIVE REVIEW | | | | | | |
| --- | --- | --- | --- | --- | --- | --- |
| Title | Year | Journal | Language | Country | Quality Indicators |  |
| Outcome metrics after burn injury from patient-reported outcome measures to Value-based health care^89^ | 2017 | Clinics in Plastic Surgery | English | USA | - gross mortality  - length of hospital stay  - quality of life |  |
| Quality assurance in burn patient care: the James Laing Memorial Essay, 1994^21^ | 1995 | Burns | English | Australia | - mortality  - length of hospital stay  - injury assessment |  |
| Measuring burn injury outcomes^20^ | 2014 | Surgical Clinics of North America | English | USA | - mortality: LA50  - length of hospital stay/total body surface area  - depression, acute stress disorder  - escharotomy, fasciotomy  - resuscitation – fluid volume/urinary output  - total calories and protein intake and weight loss or gain at discharge  - use of glutamine and oxandrolone  - mean weekly HR levels – diet markers  - physical-functional assessment in 48 hours  - time to complete removal of the eschar  - burn wound infection  - time to wound closure/ percentage of wound healed at discharge  - need for regrafting  - strength assessment, ROM  - quality of life  - documentation of injury characteristics/presence of inhalation injury |  |
| Clinical nutrition protocols for continuous quality improvements in the outcomes of patients with burns.^54^ | 1997 | Journal of Burn Care & Rehabilitation | English | USA | - determination of caloric and protein targets and intake  - micronutrient supplementation  - weekly serum levels of transferrin, pre-albumin, and daily nitrogen balance  - weight at discharge |  |
| Adult stem cells as a therapeutic option in a massive burn patient adult population with compromise of more than 50% of the body surface area^64^ | 2015 | Revista Salud Bosque | Spanish | Colombia | - mortality  - quantity of surgical procedures  - length of hospital stay |  |
| The role of arginine and glutamine in immunomodulation in burned patients - a literature review^58^ | 2015 | Rev. Brasileira de Queimaduras | Portuguese | Brazil | - incidence of infection  - length of hospital stay  - laboratory diet markers/defense system |  |
| Le estubazioni non pianificate nelle terapie intensive: quali implicazioni per l'assistenza infermieristica?^79^ | 2004 | Assistenza infermieristica e ricerca | Italian | Italy | - unplanned extubation |  |
