# Supplementary material for: Quality indicators for hospital burn care: a scoping review
Source: BMC Health Serv Res. 2024 Apr 19;24:486. doi: 10.1186/s12913-024-10980-7 (PMC11031897; doi:10.1186/s12913-024-10980-7)
Supplement: Supplementary file 4 — Supplementary Material 4. [file 12913_2024_10980_MOESM4_ESM.docx]

**Additional file 4 – Quality indicators**

**Table 1 –** Structural quality indicators

| **Human resources** | |
| --- | --- |
| Is the burn surgeon available on call 24 hours day? Is a burns theatre available on a 24-hour basis? [4] | Measures were predominately dichotomous, expressed as proportions for yes/no responses or rates of the proportion within a period of time |
| Is multidisciplinary care provided within the burns unit? [4] |  |
| **Organizational resources** | |
| Are weekly multidisciplinary team meetings conducted in the burns unit? [4] | Measures were predominately dichotomous, expressed as proportions for yes/no responses or rates of the proportion within a period of time |

**Table 2** – Process quality indicators

| **Burn patient assessment** | |
| --- | --- |
| **Evaluation by a surgeon/nurse** | |
| Was a burn surgeon or nurse practitioner assessment completed within 24 hours of admission? [4] | Measures were predominately dichotomous, expressed as proportions for yes/no responses or rates of the proportion within a period of time |
| **Wound assessment** | |
| Was there evidence in the medical history that an accepted diagram was used to accurately calculate % TBSA by the burn clinicians at the burn unit (e.g. Lund Browder or the Rule of Nine)? [2] | Measures were predominately dichotomous, expressed as proportions for yes/no responses or rates of the proportion within a period of time |
| **Multidisciplinary assessment** | |
| Did adults with a burn exceeding 15% TBSA and children with a burn exceeding 10% TBSA receive assessment of their physical functioning by a physiotherapist and/or OT within 48 h of admission? [4] | Measures were predominately dichotomous, expressed as proportions for yes/no responses or rates of the proportion within a period of time |
| For patients with a LOS exceeding 48 h, did the patient have a physical functioning assessment by the physiotherapist/OT within 48 h of admission? [2] |  |
| If the patient had a length of stay greater than 2 weeks; Were they weighed within 3–5 days of admission? Were they weighed weekly during their episode of care? [4] |  |
| Nutritional assessment. [30] | - Number of patients with weight and height recorded on admission  - Number of patients with at least one weight recording after admission  - Number of patients with a complete nutritional assessment at any time within the first 72 hours of admission |
| For patients with LOS greater than 24 h, was the patient screened for risk of malnutrition within 24 h of admission?  If the malnutrition risk screening was positive, did the patient have a complete nutritional assessment within 24 h of the positive screen? [2] | Measures were predominately dichotomous, expressed as proportions for yes/no responses or rates of the proportion within a period of time |
| Indication for artificial nutritional support. [30] | Number of patients with ≥20% TBSA with an indication for some form of artificial nutritional support (enteral or parenteral) |
| For patients with a LOS exceeding 48 h, did they have their psychosocial needs screened during their admission?  For patients who tested positive on their psychosocial screen, were they referred to psychosocial services within 24 h of the positive screen? [2] | Measures were predominately dichotomous, expressed as proportions for yes/no responses or rates of the proportion within a period of time |
| Psychology screen. [8] | In-person psychology screen in either inpatient or outpatient setting within a month of injury for admitted burn patients |
| Did the patient have a pain assessment completed (using a validated pain scale) within 24 h of admission? [2] | Measures were predominately dichotomous, expressed as proportions for yes/no responses or rates of the proportion within a period of time |
| During this admission did this patient have a positive blood culture?  If there was a positive blood culture, what organisms were grown?  Did this patient have a positive swab for infection on admission?  If there was a positive swab on admission, what organisms were grown? [4] | Measures were predominately dichotomous, expressed as proportions for yes/no responses or rates of the proportion within a period of time |
| Infection surveillance on admission. [30] | Number of patients with a lymphocyte count obtained at any time within the first 72 hours of admission |
| **Burn patient treatment** | |
| **Fluid resuscitation** | |
| For burns >20% TBSA (adults) and >10% TBSA (children): Was there evidence/documentation in the medical record, that an accepted Formula (Parklands or similar) was used to estimate the patients fluid resuscitation requirements in the first 24 h of admission? [2] | Measures were predominately dichotomous, expressed as proportions for yes/no responses or rates of the proportion within a period of time |
| To compare total volume administered and Parkland estimates, total volumes for each patient were recalculated to determine volume by weight and volume by TBSA actually administered at 24 hours and 48 hours. [46] | Average 24-hour mL/kg per %TBSA burn  Average 48-hour mL/kg per %TBSA burn |
| - Intravenous fluid intake.  - Hourly urine output on the second day post burn. [16] | - (total fluid in 24 hours in mL) / (weight x percent total burn)  - Mean urine output and the ratio of mean output to mean input |
| - 24-Hour central venous pressure.  - 48-Hour central venous pressure.  - 24-Hour lactate. [46] | Mean and standard deviation |
| **Nutritional care** |  |
| For an adult with >20% TBSA and a child with >10% TBSA was enteral or parenteral feeding commenced within 24 hours of injury? [4] | Measures were predominately dichotomous, expressed as proportions for yes/no responses or rates of the proportion within a period of time |
| If %TBSA exceeds 20% in adults and 15% in children – Was Enteral/Parenteral Nutrition commenced within 24 h of admission to the Burn Service? [2] |  |
| Enteral feeds initiated within 24 h of admission. [8] | Number of patients receiving enteral feeds within 24 h/Total number of burn patients admitted |
| Total calorie and protein intake. [20] | Intake during the first week of admission |
| Sufficient energy supply for estimated needs. [30] | Number of patients with ≥20% TBSA who receive sufficient energy supply for their current metabolic state |
| Prospective reassessment of nutritional intake. [30] | Number of patients with ≥20% TBSA who have their nutritional intake reassessed prospectively |
| Glutamine use. [20] | Yes/no |
| Indication for oral nutritional supplementation. [30] | Number of patients with TBSA between 10%–19.9%, without any other comorbidity, with an oral nutritional supplementation indication (micronutrients included) |
| Nutritional balance. [30] | Number of patients with ≥20% TBSA who have a daily nutritional balancing |
| Dietary markers – Serum albumin. [30] | Number of patients with a serum albumin value obtained within the first 72 hours of admission |
| **Surgical care** | |
| Time of arrival to initial burn wound debridement. [8] | Time in minutes admitted patients waited for initial wound debridement (hospital arrival to initial wound debridement) |
| Time to excision of burns for inpatients. [8] | Time in days from admission to the first OR excision of burn(s) (hospital arrival to excision) |
| For full thickness burns was a complete excision of the burn completed by day 5 of admission? [4] | Measures were predominately dichotomous, expressed as proportions for yes/no responses or rates of the proportion within a period of time |
| What date was the deep burns excision completed? [2] |  |
| Time to burn eschar removal. [20] | Time (days) to complete (>95%) removal of the burn eschar |
| Time from first to last graft (in days). [16] | A regression model was obtained to predict the average full thickness of burn covered for a given time from first to last graft |
| **Pain management** | |
| Time of arrival to first dose of pain medication. [8] | Time admitted patients waited in minutes before receiving pain medication (hospital arrival to first pain med) |
| **Prophylactic measures** | |
| If the patient is aged 16 years or older, did they receive anticoagulation prophylaxis? [2] | Measures were predominately dichotomous, expressed as proportions for yes/no responses or rates of the proportion within a period of time |
| - Hand hygiene adherence rate.  - Hand hygiene performed correctly rate. [73] | - Number of hand hygiene procedures performed x 100    Total observed  - Number of correct hand hygiene x 100    Total number of hand hygiene  The target value for the team's hand hygiene execution rate was ≥88%, and the target value for the correct hand hygiene rate was ≥95% |
| Implementation of preventive measures for central venous catheter-associated bloodstream infection. [73] | - Rate of implementation of preventive measures for central venous catheter- associated bloodstream infections  - Incidence of central venous catheter-related bloodstream infection |
| Fixation of the indwelling bladder catheter. [73] | - Standard fixation rate of the indwelling bladder catheter to the body surface  - Incidence of catheter-associated urinary tract infection |
| Implementation of preventive measures for VAP. [73] | - Rate of implementation of preventive measures for VAP  - Incidence of VAP |
| **Complications** | |
| Complications of resuscitation volume. [46] | Documentation of complications within 5 days of admission:  - Pulmonary edema  - Adult respiratory distress syndrome  - Abdominal compartment syndrome/ abdominal hypertension |
| Positive blood cultures:  Did this patient have any NEW positive micro results (regardless of location) of the following organisms:  - MRSA  - VRE  - Carbopenim resistant *Pseudomonas*  - Carbopenim resistant *Enterobactrer*  If yes:  - Site and date of positive swab  - Was this isolated on admission? [2] | Measures were predominately dichotomous, expressed as proportions for yes/no responses or rates of the proportion within a period of time |
| Central line–associated bloodstream infections (CLABSIs). [76] | Calculated as CLABSIs/1000 catheter days |
| Was there a negative change of >30 mL/min/1.73 m^2^ of estimated GFR (eGFR) within 72 hours of admission? [4] | Measures were predominately dichotomous, expressed as proportions for yes/no responses or rates of the proportion within a period of time |
| Was the patient readmitted to the ICU?  Date and time of readmission.  Was this readmission planned or unplanned? [2] | Measures were predominately dichotomous, expressed as proportions for yes/no responses or rates of the proportion within a period of time |
| Regrafting. [20] | Occurrence of regrafting of any autografted site |

TBSA – Total body surface area; OT – Occupational therapy; LOS – Length of stay; VAP – Ventilator-associated pneumonia; MRSA – Methicillin-resistant *Staphylococcus aureus*; VRE – Vancomycin-resistant *Enterococc*; CLABSIs – Central line–associated bloodstream infections; GRF – Glomerular filtration rate; ICU – Intensive care unit.

**Table 3** – Outcome quality indicators

| **Mortality** |  |
| --- | --- |
| Indirect standard mortality ratio (SMR). [16] | - Ratio of the observed number of deaths to the expected number of deaths  An SMR less than one for a unit indicates a better survival rate for that unit than would be expected. An SMR greater than one indicates a worse survival rate than expected for that unit after adjustment |
| **Length of hospital stay** | |
| Length of hospital stay. [20,27,99] | Length of hospitalization (days) per TBSA burn (%) |
| Overall LOS for acute episode of care and ICU overall LOS. [4] | Mean |
| The LOS, defined as the time period between the day of admission to day of discharge from ICU or burn ward based on number of nights spent in hospital. [90] | Average length of stay (ALOS) = Total length of stay/Total number of transfer outpatient |
| Mechanical ventilator time. [4] | Mean |
| **Wound healing** | |
| Graft take. [8] | Graft take at time of first dressing change as documented in surgeon note. Number of patients with graft take greater than 90%/Total number of patients requiring skin graft |
| Wound closure (to include autografting of deep burns). [20] | - Time (days) to complete (>95%) wound closure  - Size (TBSA) of open wounds at time of hospital discharge |
| **Nutritional outcomes** | |
| Did the patient lose weight during their episode of care (taken days 3–5). [4] | Measures were predominately dichotomous, expressed as proportions for yes/no responses or rates of the proportion within a period of time |
| Weight gain/loss. [20] | Admission weight (dry weight), hospital discharge weight |
| **Other result indicators** |  |
| Was the patient readmitted to the ICU?  Date and time of readmission.  Was this readmission planned or unplanned? [2] | Measures were predominately dichotomous, expressed as proportions for yes/no responses or rates of the proportion within a period of time |
| Unplanned readmission within 28 days of discharge. [4] | Measures were predominately dichotomous, expressed as proportions for yes/no responses or rates of the proportion within a period of time |

SMR – Standard mortality ratio; LOS – Length of stay; TBSA – Total body surface area.
